# AGRP neurons modulate fasting-induced anxiolytic effects

**DOI:** 10.1038/s41398-019-0438-1

**Published:** 2019-03-08

**Authors:** Changhong Li, Yanjun Hou, Jia Zhang, Guangzhi Sui, Xueliang Du, Julio Licinio, Ma-Li Wong, Yunlei Yang

**Affiliations:** 1 Department of Medicine, Albert Einstein College of Medicine, Bronx, New York USA; 2 Department of Neuroscience, Albert Einstein College of Medicine, Bronx, New York USA; 3 Department of Neurology, Beijing Haidian Hospital, Haidian Qu, Beijing PR China; 4 Department of Psychiatry, State University of New York Upstate Medical University, Syracuse, New York USA; 5 Einstein-Mount Sinai Diabetes Research Center, Albert Einstein College of Medicine, Bronx, New York USA; 6 The Fleischer Institute for Diabetes and Metabolism, Albert Einstein College of Medicine, Bronx, New York USA; 7 Department of Neuroscience, State University of New York Upstate Medical University, Syracuse, New York USA; 8 Henan Provincial People’s Hospital, Zhengzhou University, Zhengzhou, Henan China

## Abstract

Recent studies indicate that activation of hypothalamic Agouti-related protein (Agrp) neurons can increase forage-related/repetitive behavior and decrease anxiety levels. However, the impact of physiological hunger states and food deprivation on anxiety-related behaviors have not been clarified. In the present study, we evaluated changes in anxiety levels induced by physiological hunger states and food deprivation, and identified the neuron population involved. Ad libitum fed and fasted mice were tested in the open field and elevated plus-maze behavioral tests. The DREADD approach was applied to selectively inhibit and stimulate neurons expressing Agrp in hypothalamic arcuate nucleus in Agrp-Cre transgenic mice. We found that anxiety levels were significantly reduced in the late light period when mice have increased need for food and increased Agrp neurons firing, in contrast to the levels in the early light period. Consistently, we also found that anxiety was potently reduced in 24-h fasted mice, relative to 12-h fasted mice or fed ad libitum. Mechanistically, we found that chemogenetic activation of Agrp neurons reduced anxiety in fed mice, and inactivation of Agrp neurons reduced fasting-induced anxiolytic effects. Our results suggest that anxiety levels may vary physiologically with the increasing need for food, and are influenced by acute fasting in a time-dependent manner. Agrp neurons contribute to fasting-induced anxiolytic effects, supporting the notion that Agrp neuron may serve as an entry point for the treatment of energy states-related anxiety disorders.

## Introduction

Anxiety rates have been steadily increasing worldwide, presenting a significant healthcare concern. Although mechanisms controlling anxiety levels are multiple, it has been indicated that anxiety is also associated with energy states. For example, our recent studies demonstrated that energy surfeit leads to increased anxiety in diet-induced obesity (DIO) by high-fat diet (HFD) or regular chow (RC) in mice^1^, and supported previous findings that both anxiety and depression are comorbidities of obesity in human and rodents^2–7^. Recently, it has been shown that brain circuits involving hypothalamic arcuate (Arc) nucleus Agrp neurons that drive feeding and stereotypic/repetitive behaviors are distinct, they do not completely overlap, and that neuropeptide Y type 5 receptor (NPY_5_R) signaling is necessary for Agrp neuron-induced forage-related/stereotypic behaviors^8^. Agrp neurons activation was anxiolytic in several behavioral tests (two-stage open field test, zero-mazes, and plus-mazes)^8^. Furthermore, Agrp neuron stimulation decreased anxiety-related behaviors depending on food location in the two-stage open field and zero-maze tests^9^. Meanwhile, emerging evidence also indicates that hunger reduces aggressive behavior and fear in mice^10^. These results indicate that hunger states control anxiety levels bidirectionally. However, data linking circadian variations of hunger states to anxiety have not been determined.

Emerging evidence indicates that selective activation of orexigenic Agrp neurons in the Arc nucleus, a crucial brain region involved in the control of energy intake and expenditure^11^, reduces aggressive-related behaviors and inhibits anxiogenic neurons in the medial amygdala (MeA) nucleus via inhibitory GABAergic projections^10^. Put together; these findings led us test the hypotheses that: (1) anxiety levels may vary with physiological hunger states, and (2) food-deprivation exerts anxiolytic effects, which is at least partly mediated by fasting-induced activation of the orexigenic Agrp neurons in the Arc nucleus. To test this, we examined anxiety levels in food-deprived and ad libitum fed mice by using the open field and elevated plus-maze tests. We also took advantage of the Cre-loxP technique to apply chemogenetics to manipulate Agrp neurons specifically during behavioral tests. Our data support that the anxiety levels have circadian variations that correlate with physiological hunger states and are influenced by acute fasting in a time-dependent manner, and that selective chemogenetic inactivation of Agrp neurons diminishes food deprivation-induced anxiolytic effects. Collectively, our data corroborate that hypothalamic neurons that control feeding also contribute to modulating anxiety levels.

## Materials and methods

### Animals

Experimental protocols were conducted according to U.S. National Institutes of Health guidelines for animal research and were approved by both the Institutional Animal Care and Use Committees at Albert Einstein College of Medicine and State University of New York Upstate Medical University. Male and female wild-type C57/BL6J mice and Agrp-IRES-Cre transgenic mice (5–8 weeks of age, Jackson Lab^12^,) were used. Mice were maintained on a 12-h light (08:00)/dark (20:00) cycle with ad libitum access to water and mouse chow (PicoLab Rodent Diet 20, 5058, LabDiet), and group-housed 3–5 mice per cage. All animal experiments were performed in the early light period, ELP (09:00–11:00) or in the late light period, LLP (18:00–20:00) in a brightly lit room. Animals were randomized to different experimental groups. We observed no apparent differences between male and female mice; therefore, data collected from male and female mice were grouped, unless otherwise noted. Food deprivation was conducted from 21:00 to 09:00 (12-h fast), and from 09:00 to 09:00 next day (24-h fast), during which mice had water ad libitum.

### Stereotaxic viral delivery

Procedures have been detailed in our recent studies^13–15^. Briefly, Agrp-IRES-Cre transgenic mice were anesthetized with a mixture of ketamine (100 mg/kg) and xylazine (20 mg/kg), or isoflurane (3%), and placed on a stereotaxic rig (David Kopf Instruments, Tujunga, CA or Harvard Apparatus, Holliston, MA). Mouse skull was exposed via a small incision, and two small holes were drilled, one on each side of the midline for viral injections. A pulled-glass pipette with 20∼40 µm tip diameter was inserted into each side of the brain and injected (200∼300 nl in each side) with vectors AAV_2_-syn-DIO-hM3D_q_-mCherry, AAV_2_-syn-DIO-hM4D_i_-mCherry, or AAV_2_-syn-DIO-mCherry (Addgene, Cambridge, MA) in coordinates around the hypothalamic Arc nucleus (coordinates, bregma: −1.2 mm; midline: ±0.2 mm; skull surface: −5.85 mm and −5.7 mm). A micromanipulator (Narishige, Amityville, NY) was used to control viral injections at a speed of 30 nl per min, and the injection pipette was withdrawn 15 min after each injection. Mice were returned to their home cages and single housed for at least two weeks to recover. Mice were injected subcutaneously with buprenex (0.1 mg/kg twice per day) for three days to reduce postoperative pain.

### Open field test (OFT)

The procedures for the OFT were described in our recent studies^14,16^. In brief, on the day of the OFT, mice were transported to the behavioral testing room and allowed to habituate for at least thirty minutes before experiments. OFTs were performed in a brightly lit room. Mice were placed in the center of the open field, and their exploratory behaviors were tracked for 10 min and quantified using ANY-maze software (Stoelting, Wood Dale, IL). The arena was cleaned with 70 percent ethanol between trials. The time spent and distance traveled in the center zone, mean speed, and total distance traveled were analyzed for each mouse.

### Elevated plus maze (EPM)

EPM experiments were performed similarly to those of the OFT. Briefly, the EPM is an apparatus with four arms. Two of the arms are enclosed by 15 cm tall walls and denoted as “closed arms.” The other two arms do not have walls, and are denoted as “open arms,” and each mouse could see over the edge of the open arms during testing. All animals were placed in the center of the EPM in the start zone, where the arms intersect and allowed to explore for five minutes. Exploration was monitored and recorded using ANY-Maze software (Stoelting Co., Wood Dale, IL). The arena was cleaned with 70 percent ethanol between trials. The time spent and distance traveled in the open arms and closed arms, total distance traveled, and mean speed were analyzed.

### Data analysis

Unpaired Student’s *t*-tests were used to analyze differences between two groups of mice when appropriate. Differences between more than two groups of mice were analyzed by one-way or two-way ANOVA with Tukey’s or Sidak’s posthoc tests when appropriate. Homoscedasticity and normality of male and female data were statistically analyzed appropriately. Data from all the tested animals were included for analysis unless we observed unexpected abnormal behaviors, and the AgRP neurons were transduced in all the transduced AgRP-IRES-cre mice. All data were analyzed by using GraphPad Software Prism 7.0 (La Jolla, CA).

## Results

### Circadian variation of anxiety-related behaviors in mice

**Fig. 1:**
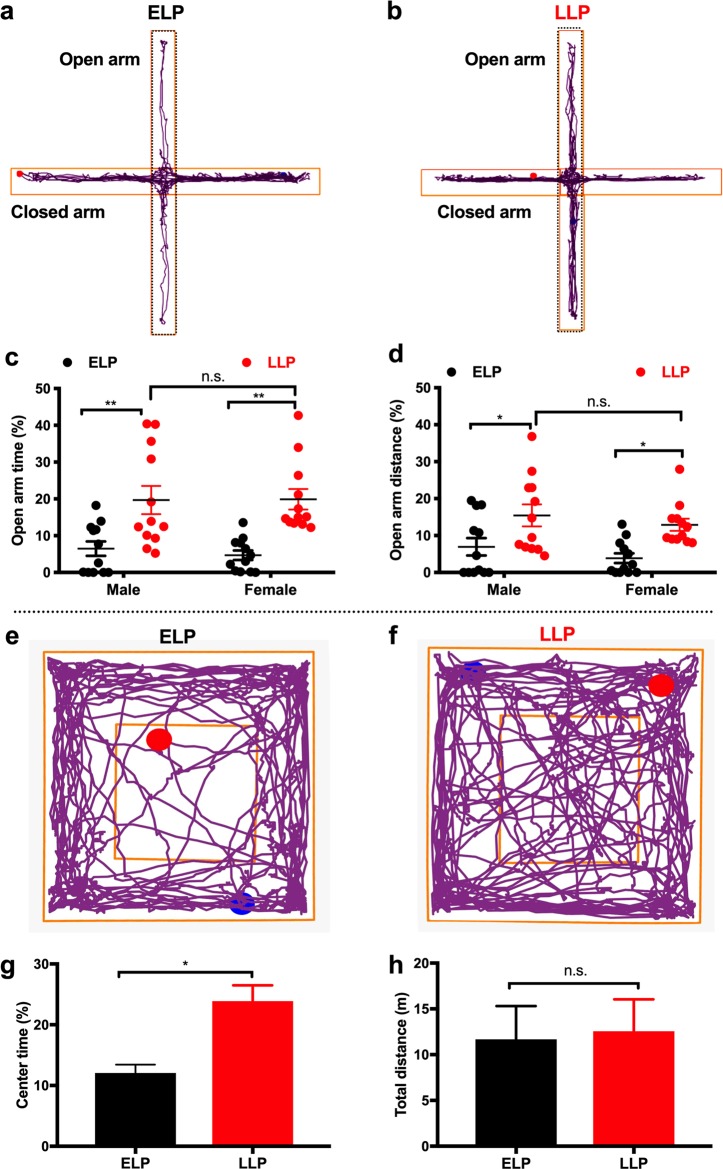
Circadian variation of anxiety levels in the early (ELP) and late light period (LLP). Elevated plus maze experiments were performed in both male and female mice in the ELP and a cohort study in the LLP. Representative traces of plus maze experiments on male mice respectively in the ELP (**a**) and the LLP (**b**). **c** Grouped data of average time spent in the open arms from **a** and **b** experiments. **d** Grouped data of open arms distance. **e–h**. Mice were tested by using open field behavioral assays in the ELP and a cohort study in the LLP. Representative traces of open field tests in the ELP (**e**) and in the LLP (**f**) (*n* = 5 male). **g** Grouped data of percent time spent in the center of the open field. **h** Grouped data of total distance traveled during the whole eperiments. Two-way ANOVA with Sidak’s *post hoc* tests were performed for **c**, **d**; Unpaired Student’s *t*-test for (**g, h**). Data represent mean ± s.e.m. ^*^p < 0.05; ** *p* < 0.01; ***p < 0.001; n.s. not significant

To define the potential differences in anxiety-related behaviors between the ELP and the LLP. Animals had ad libitum access to water and food in their home cage prior to being acclimated to the behavioral test room 30 min before EPM tests. We did not monitor when animals ate last; however, it is well-documented that mice are nocturnal animals and when fed regular chow they consume approximately 70% of their food during the dark period^17^. Interestingly, we observed that both male and female mice traveled significantly less in the “anxiogenic” open arms of the elevated plus maze in the ELP relative to a cohort group in the LLP, as indicated by the decreased time spent in the open arms (Fig. 1a–c; male mice, *n* = 12 for each group, *p* < *0.01*; female mice, *n* = 12 for each group, *p* = *0.01;* male LLP to female LLP*, p* > *0.9;* male ELP to female ELP*, p* = *0.9975;* two-way ANOVA with Sidak’s *post hoc* tests; D’Agostino-Pearson normality tests were performed using column statistics, *p* = 0.21 and 0.57 for male and female, respectively) and decreased distance traveled in the open arms (Fig. 1d; male ELP to LLP, *p* < *0.05*; female ELP to LLP, *p* < *0.05;* male ELP to female ELP*, p* = *0.9;* male LLP to female LLP*, p* = *0.96;* two-way ANOVA with Sidak’s *post hoc* tests). No significant changes were detected in total distance traveled (Supplementary Fig. 1A) and mean speed (Supplementary Fig. 1B), indicating that there were no changes in locomotion activities.

To further confirm the variation of anxiety levels, we performed the OFT in another two cohort groups. Matching the above EPM results, we observed that mice spent less time in the “anxiogenic” center area of the OFT in the ELP relative to a cohort group in the LLP (Fig. 1e–g; *n* = 5 for each group, unpaired Student’s *t*-test, *p* = *0.004*). No significant difference in total distance was detected (Fig. 1h).

### Food deprivation induces anxiolytic effects

**Fig. 2:**
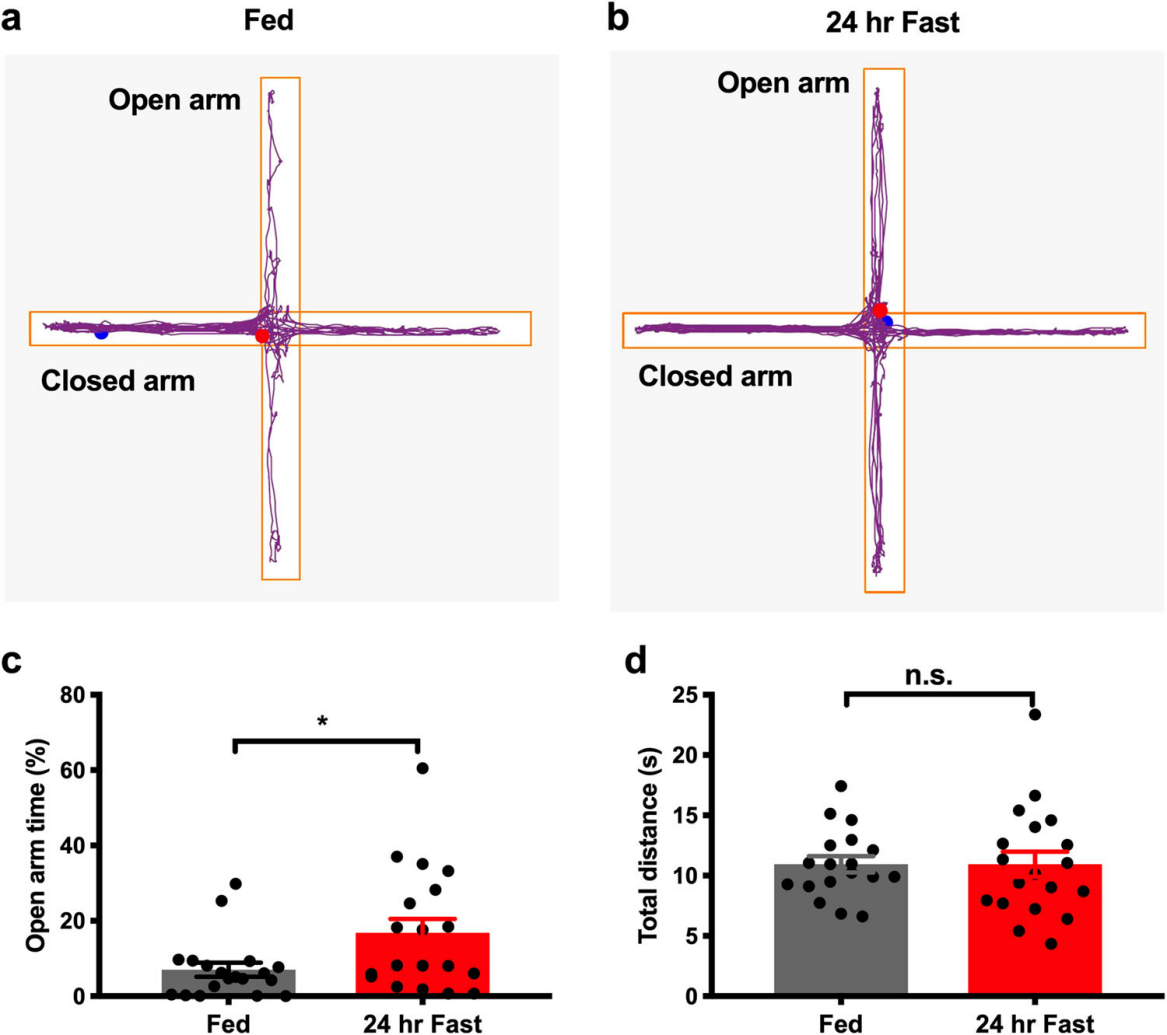
Food deprivation reduces anxiety-related behaviors in elevated plus maze tests. Individual data showing the time and distance in the open arms during the elevated plus maze behavioral tests in an ad libitum fed (**a**) and a 24-h fasted mouse (**b**). **c** Grouped data of time spent in the open arms in ad libitum fed and 24-h fasted mice. **d** Grouped data of total distance traveled in the open arms in ad libitum fed and 24-h fasted mice. Unpaired Student’s *t*-test. Data represent mean ± s.e.m. *p < 0.05; **p < 0.01; n.s. not significant; FD food deprivation

We next tested anxiety-like behaviors in fasted mice with 24-h food deprivation and in mice with free access to regular chow. EPM tests were performed for 5 min in the ELP for both fed and fasted mice. As expected, 24-h fasted mice spent more time in the open arms than ad libitum fed mice (Fig. 2a–c; *p* < 0.05, unpaired student *t*-test, for c). However, no difference was detected in the total distance traveled (Fig. 2d; *p* = 0.99, unpaired student *t*-test). Together, these results indicate that 24-h food deprivation decreases anxiety levels without affecting locomotion.

### Dependence of anxiolytic effects of food deprivation on fasting duration

**Fig. 3:**
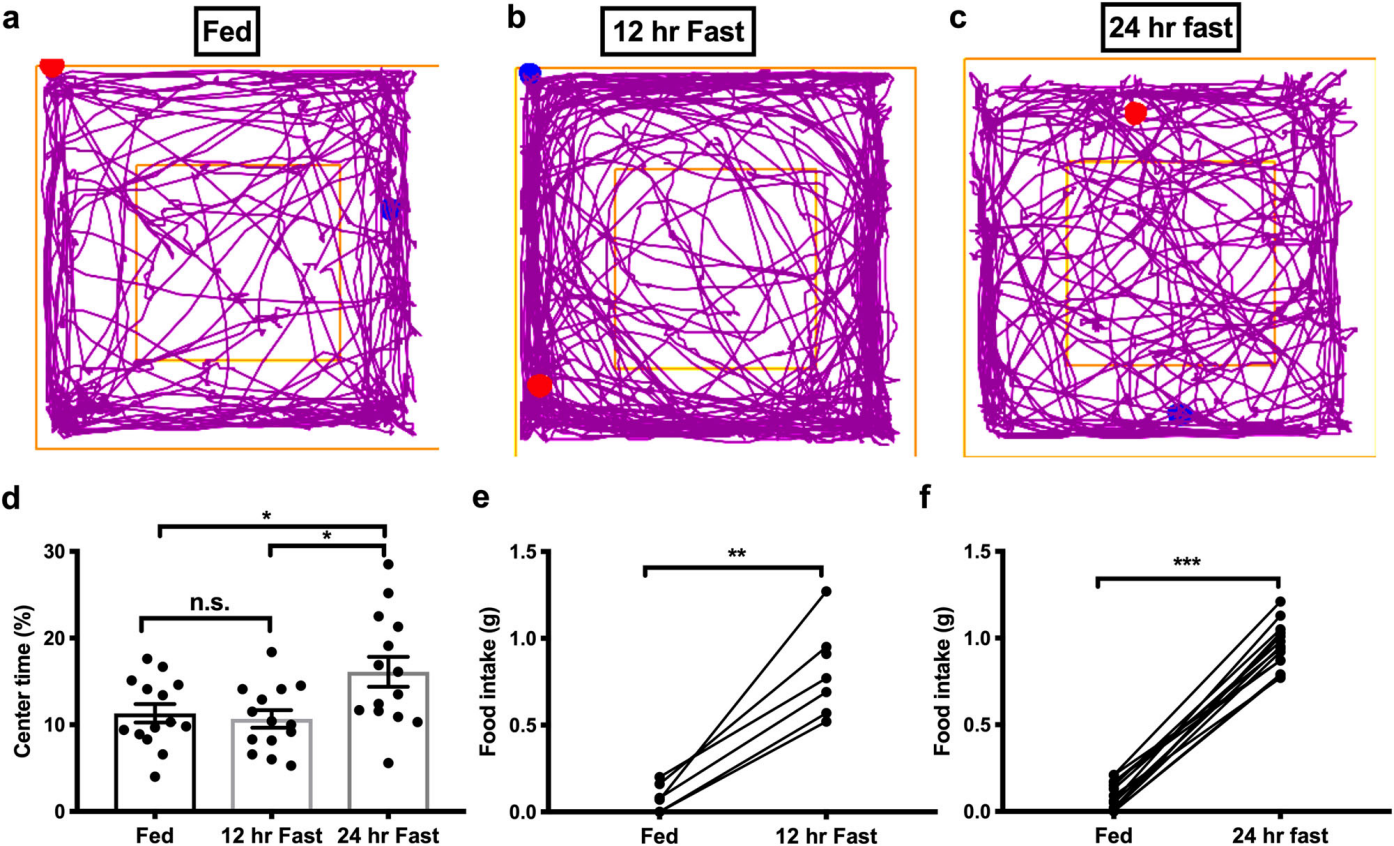
Dependence of anxiolytic effects on the duration of fasting. Representative traces of open field behavioral tests obtained from ad libitum fed mouse (**a**), 12 h fasted mouse (**b**), and 24 h fasted mouse (**c**). **d** Grouped data of average time spent in the center of the open field from *ad libitum* fed mice, and 12-h and 24-h fasted mice. **e** Two hour food intake from fed and 12-h fasted mice. **f** 2-h food intake from fed and 24-h fasted mice. One-way ANOVA with Sidak’s post hoc test was performed. Unpaired Student’s *t*-test for **e** and **f**. Data were collected from male mice and represent mean ± s.e.m. *p < 0.05; ***p < 0.001. FD food deprivation. For **a**–**c**, blue dots represent the start point, and red dots represent endpoint

We next sought to identify whether anxiety levels would be affected by fasting duration. To test this, we performed OFT in the ELP in ad libitum fed mice and those fasted for 12-h and 24-h. Interestingly, we did not observe changes in anxiety-related behaviors in 12-h fasted mice, as the time spent in the center of the open field was similar to fed mice. While 24-h fasted mice spent markedly more time in the center of the open field when compared to fed mice and to 12-h fasted mice (Fig. 3a–d; *n* = 14 for each group; fed to 12-h fasted, *p* = 0.94; fed to 2-h fasted, *p* = 0.03; 12 to 24 h fast, *p* = 0.01; one way ANOVA with Tukey’s post hoc tests). We also tested food-deprivation induced feeding behaviors. However, fasting induced a substantial increase in food intake in both 12-h and 24-h fasted mice (Fig. 3e, f).

### Agrp neurons mediate the fasting-induced reduction in anxiety levels

**Fig. 4:**
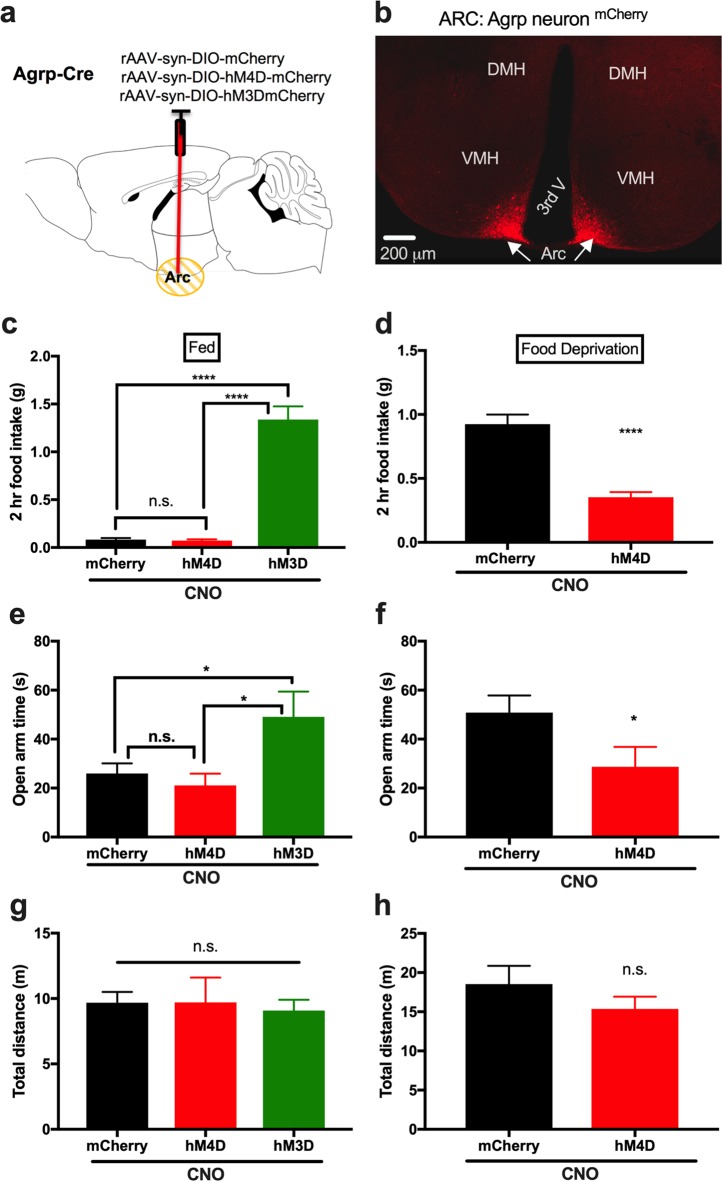
DREADD inactivation of Agrp neurons reduces fasting-induced anxiolytic effects. **a** Schematic illustration of viral injections into the hypothalamic arcuate (Arc) nucleus to achieve Cre-dependent expressions of mCherry, hM4Di, or hM3Dq in Agrp neurons in Agrp-Cre transgenic mice. **b** Representative image of transduced Agrp neurons tagged with mCherry. **c** CNO administration (i.p.; 1 mg/kg) significantly increased food intake in hM3D-transduced sated mice whereas decreased the amount of food intake in hM4D-transduced fasted mice (**d**); indicative of sufficient amount of transduced AgRP neurons for each group. **e** CNO was given 30 min before performing behavioral tests, showing that hM3D-transduced mice had increased time spent in the open arms relative to control mCherry- and hM4D-transduced sated mice. **f** CNO application decreased the time spent in the open arms in the hM4D-transduced fasted mice. CNO administration did not exert apparent effects on total distance in fed mice (**g**) and fasted mice (**h**), compared to control mCherry-transduced mice. There were no differences in normalities between male and female mice for each group by using column statistics with D’Agostino-Pearson normality tests. Data represents mean ± s.e.m. *p < 0.05; **p < 0.01; ****p < 0.0001; n.s. (not significant). DMH dorsomedial hypothalamus, VMH ventromedial hypothalamus, Arc arcuate nucleus. 3rd V third ventricle

It is well established that selective activation of Agrp neurons in the hypothalamic Arc nucleus is sufficient to rapidly induce food intake (i.e., ref. ^18^,). Moreover, our recent study^19^ and the study of Liu et al.^20^ show that the electrical firing rates of Agrp neurons are significantly increased by 24-h food deprivation. Consistently, the firing rates of Agrp neurons were significantly increased in the LLP relative to a cohort studied in the ELP^21^. Therefore, to identify the neuronal population that mediates the fasting-induced reduction in anxiety levels, we hypothethized that food deprivation-induced anxiolytic effect was mediated, at least partially, by activating Agrp neurons. This prediction was tested using a chemogenetic DREADD (Designer Receptors Exclusively Activated by Designer Drugs) approach applied in combination with the EPM test in 24-h fasted mice. DREADD technology uses engineered G protein-coupled receptors (GPCRs) that are sensitive to synthetic ligands. For instance, the engineered excitatory human muscarinic type 3 GPCR (hM3Dq) and inhibitory type 4 GPCR (hM4Di) are activated by clozapine-O-oxide (CNO)^22,23^. To investigate the potential involvement of Agrp neurons in the anxiolytic effect of food deprivation, we took advantage of viral delivery approach, as detailed in our recent studies^14,15^ and the Methods section. Bilateral injections of Cre-dependent viral vectors expressing hM3Dq, hM4Di or control fluorescent protein mCherry were performed in the Arc nucleus in Agrp-Cre transgenic mice to transduce hM3Dq, hM4Di, or control mCherry in Agrp neurons (Fig. 4a, b). We first examined AgRP neurons transduction efficiency by evaluating feeding behaviors. As expected, CNO application significantly increased food intake in fed hM3D-transduced mice (Fig. 4c; mCherry, *n* = 17; hM4D, *n* = 10; hM3D, *n* = 9; mCherry vs. hM4D, *p* = *0.99;* mCherry vs. hM3D, *p* < *0.0001;* hM4D vs. hM3D, *p* < *0.0001;* one-way ANOVA with Tukey’s post hoc tests), and decreased feeding in fasted hM4D-transduced mice relative to the control mCherry-transduced mice (Fig. 4d; mCherry, *n* = 16; hM4D, *n* = 10; mCherry vs. hM4D, *p* < 0.0001, Student unpaired *t*-test). Together, these results indicate that AgRP neurons were sufficiently transduced. We next tested the potential for AgRP neurons to affect anxiety in fed mice. CNO was applied 30 min before performing elevated plus-maze (EPM) tests. We observed that CNO application decreased anxiety in hM3D-transduced fed mice when compared to mCherry-transduced or hM4D-transduced mice (Fig. 4e; mCherry vs. hM4D, *p* = *0.85;* mCherry vs. hM3D, *p* = *0.03* < *0.01;* hM4D vs. hM3D, *p* = *0.02* < *0.01;* one-way ANOVA with Tukey’s *post hoc* test). There was no difference in the time spent in the open arms between mCherry-transduced and hM4D-transduced fed mice (Fig. 4e), which may be explained by the fact that AgRP neurons are silenced in fed conditions in the ELP^19,21^. EPM behavioral tests were performed on cohort groups of 24-h food-deprived mCherry-transduced or hM4D-transduced mice to test whether AgRP neuron inactivation reduces fasting-induced anxiolytic effects. Interestingly, we observed that the time spent in the open arms in hM4Di-transduced mice was less than that in control mCherry-transduced mice (Fig. 4f; mCherry vs. hM4D, *p* = *0.02* < *0.01;* student unpaired *t* test). There were no differences in total distance traveled between mCherry-, hM4Di- and hM3Dq-transduced fed mice (Fig. 4g), and between mCherry-transduced and hM4D-transduced fast mice (Fig. 4h).

## Discussion

Anxiety disorders represent a major public health concern as their rates have been increasing in Western societies. The causes of anxiety disorders are multiple; therefore, it is important to understand the physiological variations in energy states and anxiety, and mechanisms involved. In this study, we found that food deprivation led to decreased anxiety levels. For example, we found the mice are naturally less anxious toward the end of their light period than at the beginning of their light period. However, feeding and anxiety levels show dynamic changes in opposite directions, suggesting that anxiety levels are under circadian control in a manner that maybe negatively correlated with hunger states. In vivo single neurons spiking activity findings show that the firing rate of Agrp neurons in awake mice increase throughout the light phase. This suggests that their activity signals increasing need for food. Agrp neurons show a sudden decrease in spiking minutes after feeding starts^21^ and mice have a circadian feeding pattern even under continuous light conditions^24^, which gave the rationale to investigate whether acute fasting influenced anxiety levels. Conversely, it is noteworthy to mention other circadian patterns that could influence the Agrp neuron firing, such as the circadian rhythm of plasma glucose and insulin concentration. Glucose level increases in the beginning of the light phase, peaks in the middle of the light phase, then decreases until the beginning of the dark phase, after which it stays at basal level or has a lower peak before dawn^25–27^. Insulin level has a nocturnal peak at or just around the transition from the light to the dark phase^25,26,28^. Food intake and metabolism are closely associated with the hypothalamic-pituitary-adrenal (HPA) axis activity, which is of low amplitude at the beginning of the light phase and high amplitude at the beginning of the dark phase^29^, around the time in which peripheral corticosterone level peaks. This peak is followed by a gradual decrease throughout the dark phase, and it stays at basal levels during the light phase^28,30–34^.

We found that 24-h food deprivation significantly reduced anxiety-related behaviors in mice (Figs. 3, 4). Meanwhile, 12-h fasted mice did not show apparent changes in anxiety levels when compared to non-fasted ones (Fig. 3), suggesting that the change in anxiety levels depends on the duration of fasting. Fasting, a catabolic metabolic state, causes several physiological changes in mice (see ref. ^35^ for a review). Changes in metabolic parameters including glucose and insulin levels, which are significantly and proportionally decreased to the increase of length of fasting^36–38^, and triglyceride and free fatty acid levels are proportionally increased^36^. The stress response to fasting results in activation of the HPA axis, increasing corticosterone^34,39^, and the stimulation of the sympathetic nervous system^40^. Several hormones are also affected by fasting; for instance, leptin level is decreased proportionally to fasting duration until a stable low leptin level can be reached after 6–12 h of fasting in mice^36,41–43^. Ghrelin and neuropeptide Y levels were significantly increased, while resistin was significantly decreased by 48 h fasting^44,45^. Thyroxine (T4) and triiodothyronine (T3) levels were significantly decreased by 16–24 h fasting^36^, which can contribute to energy conservation. Testosterone levels were also significantly decreased by 16–48 h fasting^36,44^. Thus, several hormonal and metabolic changes are proportionally affected by the length of fasting, which may directly interact with Agrp neurons.

In this present study, we found that hypothalamic orexigenic Agrp neurons mediate the fasting-induced reduction in anxiety levels. Our data are compatible with those of Dietrich et al.^8^ and Burnett et al.^9^, who used modified OFT or zero-maze and EPM tests by inserting a novel object on the tests to induce novelty exploration and anxiety^46^. In our experiments, we induced anxiety by providing a bright light environment during those tests. Our data are also compatible with those of Towers et al.^47^, which show that 24-h fasting decrease anxiety-like behavior in the elevated zero maze. Furthermore, we showed that the anxiolytic effect of fasting is time dependent, as it does not occur in 12 h fasted mice. Agrp neurons activation was anxiolytic in several behavioral tests (two-stage open field test, zero- and plus-mazes). Furthermore, Agrp neuron stimulation has been reported to decrease anxiety-related behaviors depending on food location in the two-stage open field and zero maze tests^9^.

We recently found that anxiety levels were increased in obese mice which showed increased body weight and impaired glucose tolerance tests^1^. Together, these results suggest that the anxiety levels are positively correlated to energy states, which means that energy surfeit exerts anxiogenic effects and food deficit induces anxiolytic effects. Obesity is a multifaceted disorder with pathological effects beyond altered energy metabolism, which increasing rates present a significant public health concern^48^. Interestingly, it has become increasingly clear that obesity is comorbid with psychiatric disorders, including increased anxiety in both human and rodents^2–6^. Meanwhile, we have recently demonstrated a bidirectional relationship between HFD feeding and anxiety. For example, we found that HFD promotes anxiety in DIO in mice but not in diet-resistant mice^1^. Interestingly, the anxiogenic effects seen during chronic HFD feeding are also seen in regular chow feeding^1^. Thus, we proposed that HFD does not induce anxiety-related behaviors per se; HFD feeding promotes the development of both metabolic and psychiatric phenotypes, including decreased glucose tolerance and increased anxiety. However, the precise cellular and behavioral interactions that mediate the relationship between obesity and anxiety remain unclear, which require further studies.

It is well-established that hypothalamic neural circuits play crucial roles in the control of energy states. Interestingly, emerging evidence indicates that these circuits are also involved in the control of emotion-related behaviors^49–51^. Therefore, the effect of energy states on anxiety levels may be at least in part due to hypothalamic feeding neurons dysfunction. Our data indicate that Agrp neurons in the hypothalamus mediate food deprivation-induced reduction in anxiety levels, as evidenced by the fact that chemogenetic inactivation of Agrp neurons blocked deprivation-induced anxiolytic effects (Fig. 4). Meanwhile, we cannot exclude the involvement of other brain regions, including the ventral hippocampus and amygdala, that may underlie food deprivation-induced decrease in anxiety^52,53^. Further studies are needed to determine neural circuits and mechanisms governing the complex and bidirectional relationship between energy states and anxiety-related behaviors. Future studies are necessary to examine causal links between energy states and anxiety phenotypes as well as examining neural circuits that may explain the anxiety behavior phenotypes in mild, moderate, and severe food deficit conditions. As indicated in Fig. 3, there was no signifincant changes in anxiety levels in 12-h food deprived mice; however, food intake was potently and siminarly increased in 12-h and 24-h fasted mice. These indicate that 12-h and 24-h food deprivation induces similar feeding behavior but duration-dependent anxiolytic effects, indicating that 12-h food deprivation could activate Agrp neural circuits primarily for feeding while 24-h fasting activate Agrp neural circuts for both feeding and emotion-related behaviors, which is supported by the heterogeneous behavioral characteristics of Agrp neurons^54^. Thus, cellular and circuit mechanisms responsible for fasting-induced anxiolytic and fasting-increased appetite may be distinct or only partially overlapping, which need to be further studied in the future. Based on our results and literature, we propose that Agrp neurons play roles in both homeostatic feeding behaviors and hunger-induced anxiolytic effects, although we cannot exclude the involvement of other signaling pathways.

## Supplementary information

Supplemental legend

Supplemental Figure 1
