# Supplementary material for: AGRP neurons modulate fasting-induced anxiolytic effects
Source: Transl Psychiatry. 2019 Mar 8;9:111. doi: 10.1038/s41398-019-0438-1 (PMC6408535; doi:10.1038/s41398-019-0438-1)
Supplement: Supplementary file 1 — Supplemental legend [file 41398_2019_438_MOESM1_ESM.docx]

**Supplementary Figure 1: There was no difference in physical locomotion between ELP and LLP.** (A) Grouped data of total distance traveled in experiments performed in text Figure 1 A and B. (B) Mean speed during the experiments in text Figure 1A and B. Two-way ANOVA with Sidak’s *post hoc* tests were performed for each grop; data represent mean ± s.e.m. n.s., not significant.
