# Supplementary figures and images for: AGRP neurons modulate fasting-induced anxiolytic effects
Source: Transl Psychiatry. 2019 Mar 8;9:111. doi: 10.1038/s41398-019-0438-1 (PMC6408535; doi:10.1038/s41398-019-0438-1)

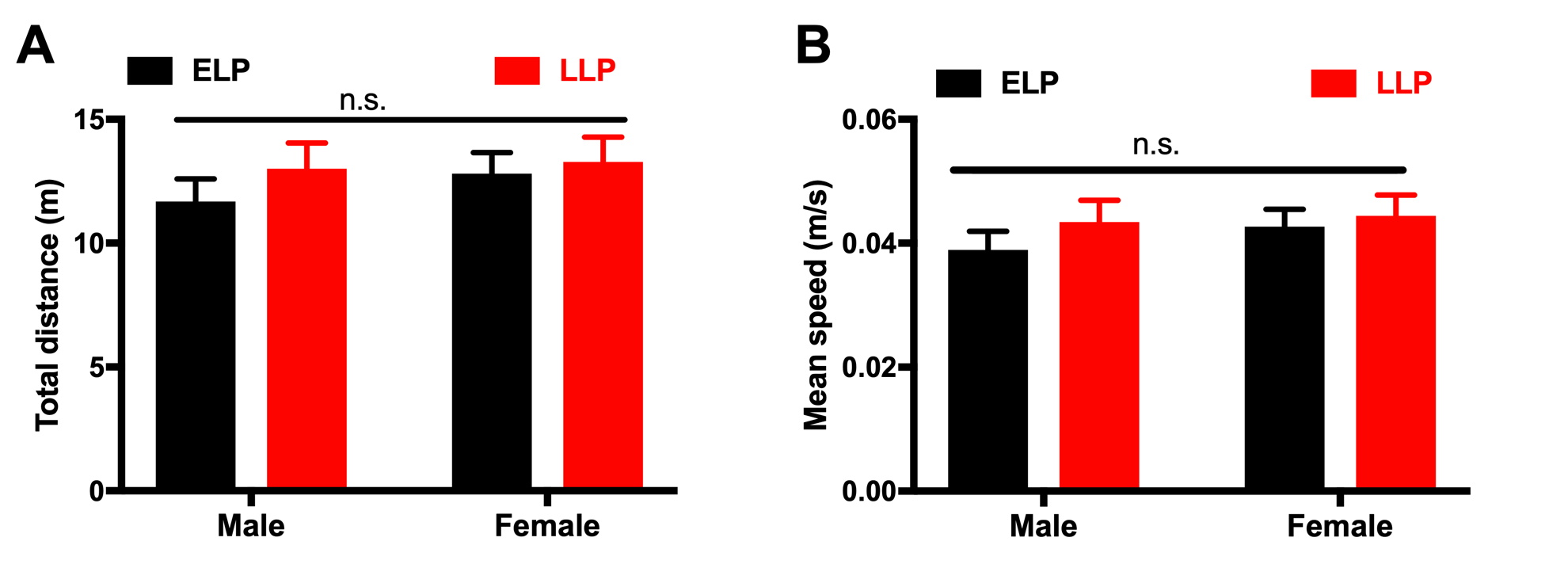

Supplement: Supplementary file 2 — Supplemental Figure 1 [file 41398_2019_438_MOESM2_ESM.tif]
